# Heavy iron in large gem diamonds traces deep subduction of serpentinized ocean floor

**DOI:** 10.1126/sciadv.abe9773

**Published:** 2021-03-31

**Authors:** Evan M. Smith, Peng Ni, Steven B. Shirey, Stephen H. Richardson, Wuyi Wang, Anat Shahar

**Affiliations:** 1 Gemological Institute of America, New York, NY 10036, USA.; 2 Earth and Planets Laboratory, Carnegie Institution for Science, Washington, DC 20015, USA.; 3 Department of Geological Sciences, University of Cape Town, Rondebosch 7701, South Africa.

## Abstract

Subducting tectonic plates carry water and other surficial components into Earth’s interior. Previous studies suggest that serpentinized peridotite is a key part of deep recycling, but this geochemical pathway has not been directly traced. Here, we report Fe-Ni–rich metallic inclusions in sublithospheric diamonds from a depth of 360 to 750 km with isotopically heavy iron (δ^56^Fe = 0.79 to 0.90‰) and unradiogenic osmium (^187^Os/^188^Os = 0.111). These iron values lie outside the range of known mantle compositions or expected reaction products at depth. This signature represents subducted iron from magnetite and/or Fe-Ni alloys precipitated during serpentinization of oceanic peridotite, a lithology known to carry unradiogenic osmium inherited from prior convection and melt depletion. These diamond-hosted inclusions trace serpentinite subduction into the mantle transition zone. We propose that iron-rich phases from serpentinite contribute a labile heavy iron component to the heterogeneous convecting mantle eventually sampled by oceanic basalts.

## Teaser

Isotopically heavy metallic iron in sublithospheric diamonds reveals deeply subducted serpentinized oceanic peridotite.

## INTRODUCTION

Global subduction of oceanic lithosphere is a fundamental characteristic of terrestrial plate tectonics. An integral feature of this recycling is that oceanic lithosphere interacts with seawater over millions of years before it subducts into the mantle, having profound implications for the geochemical cycle of water and other volatiles. Seawater circulation in faults associated with ridges, transforms, and slab bending (*1*) before subduction leads to variable but widespread, penetrative serpentinization of lithospheric mantle peridotite (*2*, *3*). The serpentinized portion of subducting slabs, especially within cool slab interiors, is recognized as a way to carry surficial materials beyond arcs and recycle them deeply into the convecting mantle (*2*, *4*–*9*). However, the signature of the serpentinized peridotitic portion of these recycled slabs has only been inferred indirectly, based on the geochemistry of oceanic basalts, erupted at ocean islands and mid-ocean ridges. Such basalts contain water and halogens (*4*), as well as noble gases (*8*), that suggest that their mantle sources are marked by extensive long-term subduction of serpentinized peridotite.

**Fig. 1:**
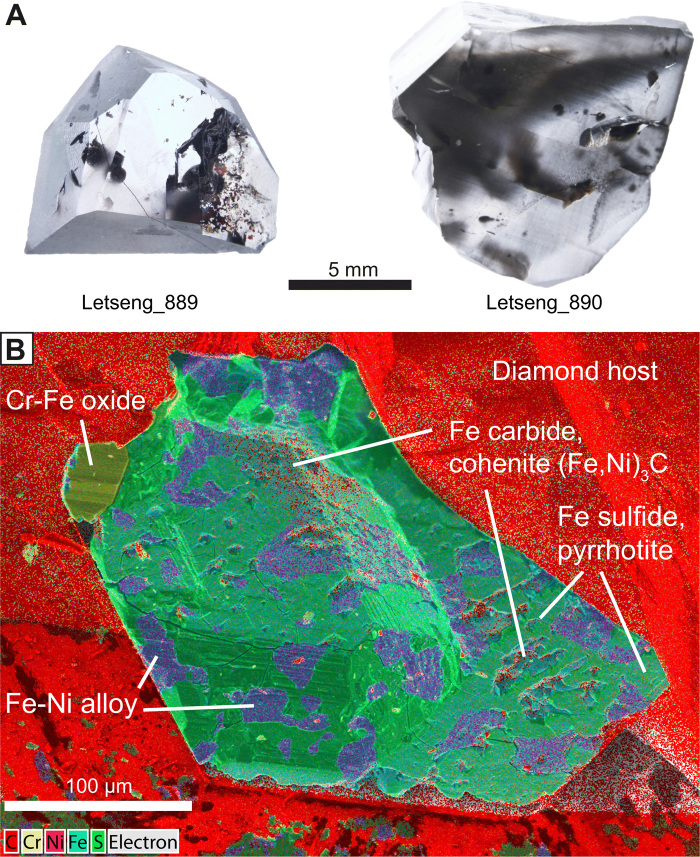
Inclusion-bearing CLIPPIR diamond samples. (**A**) Letseng_889 (2.39 carats) and Letseng_890 (6.52 carats) are offcuts from larger rough diamonds from the Letseng mine, Lesotho. These contain the pristine metallic Fe-Ni-C-S inclusions that were targeted for iron and osmium isotopic analyses. Visible black spots are graphitized decompression cracks associated with inclusions. (**B**) False-color EDS (energy-dispersive x-ray spectroscopy) map of metallic inclusion K in Letseng_889. The diamond host was cleaved (not polished) to expose this inclusion for osmium isotopic analysis, meaning that the inclusion’s exterior morphology is preserved in this view.

Insight into this recycling emerges from our iron isotopic analyses of metallic inclusions in a recently recognized variety of sublithospheric diamonds (Fig. 1A), which were undertaken to better understand the iron isotopic composition of the deep mantle and the source of the metallic inclusions. These diamonds are almost exclusively type IIa [having minimal nitrogen contents, <5 parts per million (ppm)] and form large and highly pure gems, such as the 3106-carat Cullinan diamond (*10*). These so-called CLIPPIR diamonds (Cullinan-like, large, inclusion poor, pure, irregular, resorbed) originate from depths of about 360 to 750 km, overlapping the mantle transition zone and uppermost lower mantle (*10*, *11*). The composition of their rare silicate inclusions of majoritic garnet and inferred Ca silicate perovskite as well as their carbon isotopic compositions (δ^13^C = −26.9 to −3.8‰) link CLIPPIR diamond source fluids to subducted slabs (*10*). Multiphase inclusions of metallic Fe-Ni-C-S melt are the most abundant material trapped in these diamonds (*10*). The trapped melt solidifies to an assemblage of Fe carbide (cohenite), Fe-Ni alloy, and Fe sulfide (pyrrhotite), with minor amounts of O-, Cr-, and P-bearing phases as well as an exsolved fluid phase of methane and hydrogen (Fig. 1B) (*10*). An approximate bulk composition was previously estimated to be 60 to 80% Fe, 8 to 15% Ni, 10 to 16% C, and 4 to 14% S (atomic percent), based on the relative areas of these phases in inclusion cross sections (*10*, *11*).

Here, we show that the iron isotopic compositions of these inclusions are exceptionally heavy (δ^56^Fe = 0.79 to 0.90‰, where δ^56^Fe is the parts per mil deviation of the ^56^Fe/^54^Fe ratio relative to the IRMM-014 standard) and are best explained if the iron is derived from magnetite and/or Fe-Ni alloys precipitated during serpentinization of the peridotitic mantle portion of slabs before deep subduction. This finding specifically traces a hidden but common geochemical pathway produced by subduction. The measurements also have implications for mantle iron isotope systematics. Subduction recycling of heavy iron in the form of iron-rich and relatively fusible phases could help explain the longstanding problem of the variable and heavy iron isotopic signatures of oceanic basalts (δ^56^Fe ≈ 0.1‰) relative to mantle peridotites and chondrites (both near 0.0‰) (*12*–*15*) by adding to the effects of subduction-related recycling of pyroxenite (*16*, *17*).

## RESULTS

### Iron isotope measurements

**Fig. 2:**
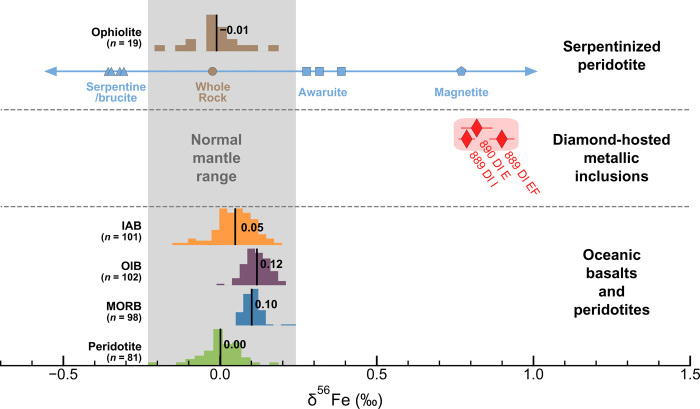
Iron isotope measurements. Metallic inclusions in diamond samples Letseng_889 and Letseng_890 are heavier than unaltered mantle-derived peridotites and basalts (*12*–*14*, *16*, *47*, *55*–*63*) but are in line with awaruite/magnetite from serpentinized peridotite (*31*). IAB, island arc basalt; OIB, ocean island basalt; MORB, mid-ocean ridge basalt.

Two gem-quality, type IIa CLIPPIR diamonds (Fig. 1A), studied previously for their inclusion mineralogy (*10*), were analyzed here for iron isotopes (Fig. 2, Materials and Methods, and tables S1 and S2). The diamonds are from the 90-million-year-old Letseng kimberlite in Lesotho (*18*). Three iron isotopic compositions were obtained from three inclusion samples, each weighing less than 4 μg (smaller than a cube of 80-μm side length), by miniaturizing chemical separation and mass spectrometric procedures. Careful measures were taken to isolate the inclusions from anthropogenic iron contamination during analysis and to select inclusions judged as pristine by their intact methane jackets, which would not be preserved if the inclusions had been altered (Materials and Methods and fig. S1). Two measurements were made from diamond sample Letseng_889, one from a co-analyzed inclusion pair and another from a separate inclusion, followed by a third isotopic measurement from a single inclusion in diamond sample Letseng_890.

Inclusions in Letseng_889 gave δ^56^Fe values of 0.899 ± 0.020‰ and 0.787 ± 0.013‰, while Letseng_890 gave 0.819 ± 0.025‰ (Fig. 2). These values are notably heavy compared to the entire −0.1 to +0.2‰ range of δ^56^Fe in oceanic basalts and unaltered mantle peridotites thought to represent the ambient convecting mantle (Fig. 2) (*13*, *14*).

### Osmium isotope measurements

**Fig. 3:**
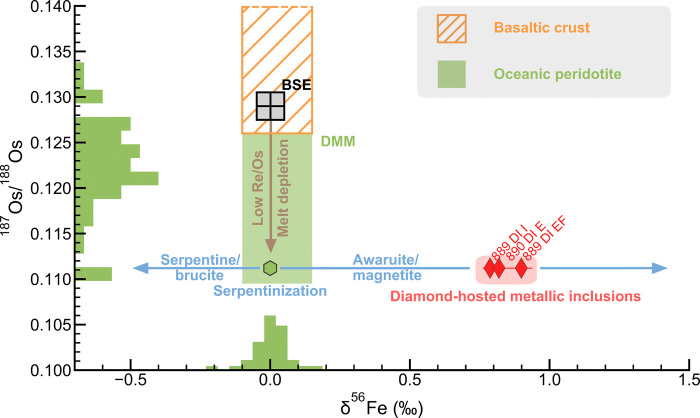
Iron and osmium isotope systematics. Inclusions in Letseng_889 and Letseng_890 have high δ^56^Fe values and low ^187^Os/^188^Os ratios that are jointly explained as originating in serpentinized oceanic peridotite. Histogram for δ^56^Fe from Fig. 2. Histogram for ^187^Os/^188^Os is for Zealandia peridotites (*37*) with Pd/Ir < 0.3, chosen to show the variability of inherited refractory components in tectonically exhumed oceanic lithospheric peridotite. BSE, bulk silicate Earth; DMM, depleted MORB mantle.

As an additional isotopic constraint on the origin of the metallic Fe-Ni-C-S inclusions, osmium isotopic compositions were measured from two separate inclusions in diamond sample Letseng_889 (Materials and Methods). The inclusions were of similar size and mineralogy (Fig. 1B) to those used for iron isotopic analysis and gave highly unradiogenic ^187^Os/^188^Os ratios of 0.1115 ± 2 and 0.1109 ± 2 (Fig. 3). These values yield Neoarchean rhenium depletion model ages (*T*_RD_) of about 2.5 Ga. While Re and Os concentrations are not reported for practical reasons (Materials and Methods), these extremely low Os isotopic compositions, by their nature, imply such a low time-averaged Re/Os that corrections for differential ingrowth of radiogenic Os since emplacement of the Letseng host kimberlite at 90 Ma are inconsequential relative to model age uncertainties.

## DISCUSSION

### Provenance of the heavy iron

Fractionation factors are generally muted at high temperatures, making it difficult to produce large isotopic shifts by crystal chemical or igneous processes in the deep mantle, though some have been suggested. Our previous petrographic and compositional study (*10*) of these metallic inclusions hypothesized that the metallic iron (Fe^0^) was created by Fe^2+^ disproportionation (3Fe^2+^ ➔ 2Fe^3+^ + Fe^0^) within Fe^3+^-bearing silicates, a process thought to produce widespread metal saturation in the mantle at depths greater than 250 ± 30 km (*19*, *20*). However, experimental results indicate that metallic iron exsolved due to disproportionation will be isotopically light (*21*–*23*), whereas the inclusions measured in this study are remarkably heavy. Therefore, the new isotopic data rule out disproportionation alone, because it is unable to account for the heavy iron signature. If metal created by disproportionation was allowed to reequilibrate at lower pressures, in the absence of Fe^3+^-bearing silicates, experiments at 1 GPa show a reversal in the sense of fractionation (enhanced by Ni or S in the metal) that would shift the metal toward heavier isotopic compositions (*24*). Yet, under the most favorable conditions for a Fe-Ni-C-S melt with a composition comparable to the inclusions, the fractionation (Δ^56^Fe_metal-silicate_) can be estimated to be less than +0.4‰ (*24*), which is relatively modest compared to our values (Supplementary Discussion).

Other high pressure-temperature processes such as core formation (*23*, *25*), partial melting, or magmatic differentiation (*22*, *26*) are also unable to explain a δ^56^Fe signature as heavy as those of the metallic inclusions in CLIPPIR diamonds (0.79 to 0.90‰) based on theory, experiments, and natural samples. Iron isotope fractionation factors are simply not big enough at these high temperatures to produce the requisite isotopic shifts from a near-chondritic mantle (0‰; Supplementary Discussion). Although the high-temperature gradient at the core-mantle boundary may conceivably produce a suitably heavy δ^56^Fe signature by thermodiffusion (Soret effect) (*27*), the core-mantle boundary is so far removed (>2000 km), spatially and geochemically, from CLIPPIR diamond growth in concert with subducted slabs at 360- to 750-km depths (*10*, *11*) that deriving a dense iron phase from the core-mantle boundary is implausible (Supplementary Discussion).

A more geologically plausible scenario involves a low-temperature fractionation mechanism that can easily generate large isotopic shifts, operating near Earth’s surface where subducting slabs originate. The most common low-temperature process on Earth affecting iron-bearing silicate and oxide minerals is the widespread seawater-induced serpentinization of the mantle rocks in the oceanic lithosphere. Faulting related to ridges, transforms, and slab bending (*1*) before subduction allows seawater to penetrate into the ocean floor, leading to an estimated 10 to 30% serpentinization in the uppermost 10 km of lithospheric mantle peridotite (*2*, *3*, *8*). Serpentinization is a common, widespread, mineralogically complex, and unevenly distributed hydrothermal alteration process affecting olivine-rich ultramafic rocks, below ~400°C. In general, water reacts with the olivine (Fo_90-92_), as well as orthopyroxene and clinopyroxene, in peridotite (~7 to 8 weight % Fe) to form the secondary minerals serpentine, brucite, and magnetite, among other secondary phases (*28*). Magnetite (Fe_3_O_4_) is the dominant iron-rich secondary mineral, although iron partitioning between (and modal abundances of) serpentine, brucite, and magnetite can vary (*29*). Multiple factors, such as the water/rock ratio, degree of serpentinization, and temperature, determine the actual reactions occurring at any time. Some of the Fe^2+^ from primary minerals oxidizes to Fe^3+^ in magnetite and serpentinite by reacting with water, which generates hydrogen and leads to an enormous reduction potential (*28*, *29*). The hydrogen (H_2_) lowers the oxygen fugacity such that Fe-Ni alloys (e.g., awaruite) often precipitate from the fluid in partially serpentinized peridotite (*28*, *30*).

The iron-rich secondary phases precipitated during serpentinization, magnetite and Fe-Ni alloys, have a heavy iron isotopic composition (*31*). For example, heavy iron in magnetite (0.77‰) and awaruite (0.28 to 0.39‰) has been measured in partially serpentinized peridotites from New Caledonia (*31*). Relatively strong bonding of either Fe^3+^ in magnetite or Fe^0^ in Fe-Ni alloys accounts for the fractionation of heavy iron into these phases relative to the lighter iron in serpentine and brucite or in aqueous complexes (*31*). Strong positive iron isotopic fractionation between these phases and iron-bearing fluid complexes at low temperatures during serpentinization are also supported by nuclear resonant inelastic x-ray scattering (NRIXS) data and ab initio calculations (fig. S2). Furthermore, magnetite formed in experiments equilibrating magnetite and fayalitic olivine (*22*), or in other surficial settings at low temperatures, such as banded iron formations, also tends to be isotopically heavy, although a range is evident (*14*).

These iron-rich phases in serpentinite could be subducted down to the mantle transition zone to provide the heavy iron signature observed in CLIPPIR diamonds. During subduction, at relatively shallow levels in the mantle wedge/arc system, serpentinites can expel fluid that preferentially carries away light iron and increases the bulk rock δ^56^Fe composition of residual serpentinites in the slab by as much as 0.15‰ (*32*). However, although this shallow fluid loss apparently strengthens the attribution of a high δ^56^Fe signature to serpentinite, it is likely not relevant for CLIPPIR diamonds. Fluid evolution in this process removes carbon, hindering later diamond growth, and produces variably oxidizing conditions (*32*, *33*) that would be difficult to reconcile with the reduced nature of the metallic inclusions studied here. Such shallow devolatilization typically affects hotter slabs, whereas CLIPPIR diamonds are thought to be associated with the deep subduction of cooler slabs, whose partially serpentinized interiors remain hydrated to great depths, reaching the mantle transition zone (*5*–*7*) with their carbon budget intact. This deep, cold slab subduction is a separate pathway that largely bypasses the dehydration of the mantle wedge/arc system (*9*, *34*, *35*).

Additional evidence to trace the provenance of this heavy iron is provided by osmium isotopic compositions, which unambiguously connect them to peridotitic lithologies. The two additional metallic inclusions in sample Letseng_889 that were analyzed for their ^187^Os/^188^Os ratio gave values of 0.1115 ± 2 and 0.1109 ± 2 (Fig. 3). These are extremely low and unradiogenic relative to the composition of the present-day ambient convecting mantle that would be inferred from the osmium isotopic composition of oceanic basaltic volcanism (~0.127 to ~0.135). However, unradiogenic osmium isotopic compositions are commonly found in abyssal peridotites (*36*), tectonically exhumed peridotites (*37*), and mantle xenoliths in ocean island basalts [(*38*) and data therein] where they are interpreted as ancient signatures (*39*) that are simply relicts of melt depletion related to previous cycles of oceanic mantle convection (*36*, *40*). Such unradiogenic osmium is not found in basaltic crust or sediments (including banded iron formations) (*40*). Unradiogenic osmium signatures are typically hosted in ultratrace abundance refractory Os-Ir alloys that can be associated with awaruite (*30*) and would have such high osmium concentrations that they would remain unaffected by any radiogenic seawater osmium during serpentinization. The combined iron and osmium constraints suggest that the observed heavy δ^56^Fe is a product of serpentinization in oceanic peridotite, where the low ^187^Os/^188^Os signature is an inherited relict from prior mantle convection (Fig. 3).

**Fig. 4:**
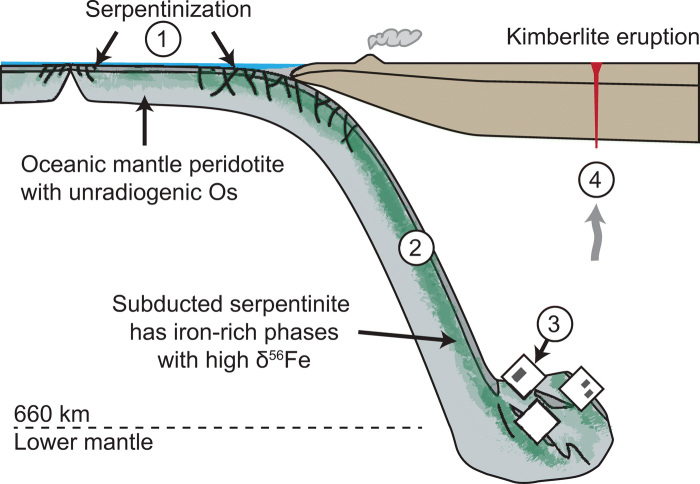
Heavy iron subduction. At left, a mid-ocean ridge produces basaltic crust, while the underlying oceanic peridotite residue has low Re/Os ratios and time-integrated unradiogenic Os (variably low ^187^Os/^188^Os) from prior mantle convection and melting. (1) Serpentinization at mid-ocean ridges, transforms, and bending-related faults leads to enrichment of isotopically heavy iron in magnetite and Fe-Ni alloys. (2) Subduction conveys these iron-rich phases to the transition zone/lower mantle. (3) Fe-Ni-C-S metallic melt with high δ^56^Fe and unradiogenic Os is trapped as inclusions in large gem diamonds (CLIPPIR diamonds) such as those examined in this study. (4) Upward transport of diamonds by localized buoyancy associated with diamond formation or an external mechanism such as a plume, with ultimate exhumation to surface via kimberlite volcanism.

The iron within the metallic inclusions in diamond is therefore proposed to be sourced from the isotopically heavy, iron-rich phases in serpentinized peridotite, either magnetite or Fe-Ni alloys (Fig. 4). Derivation from magnetite, an iron oxide, would require an additional step to reduce the iron to account for the reduced, metallic nature of the Fe-Ni-C-S inclusions (*10*). Reducing fluids arising from H_2_ generated by serpentinization may provoke this reduction, either during seafloor alteration or during early stages of subduction (*28*, *41*). Reduced fluids containing both H_2_ and CH_4_ in association with magnetite and Fe-Ni alloy are observed in olivine-hosted fluid inclusions (*42*). Alternatively, the iron in the diamond-hosted inclusions could be sourced from Fe-Ni alloys precipitated during serpentinization. Although the Fe-Ni alloy typically reported in serpentinite is awaruite (Ni_2-3_Fe), which is more nickel-rich than the observed Fe-Ni-C-S inclusions, the sparse data on metallic phases in serpentinites show that their compositions can vary and that other alloys, such as native Fe, can also form during serpentinization depending on the conditions (*28*, *41*).

Fe-Ni alloy precipitation occurs during serpentinization in multiple oceanic settings (*28*, *30*). For example, awaruite is common throughout brucite-bearing partly serpentinized peridotites observed at the slow-spreading mid-Atlantic ridge (*43*, *44*). Notably, bending-related faults at the outer rise of subduction zones (*1*) could be favorable sites for serpentinization with localized deposition of Fe-Ni alloys reaching deep into the slab, because the low water/rock ratios expected there correspond experimentally to the most reducing conditions (*45*). Serpentinites from this setting, however, have not been directly studied in detail due to their inaccessibility. At least one ophiolite locality demonstrates that, under favorable conditions, fault-localized reducing fluids can yield Fe-Ni alloy nuggets (josephinite) sometimes reaching several kilograms (*28*).

Although it is presently unclear whether the iron in Fe-Ni-C-S inclusions comes from magnetite or Fe-Ni alloys, the origin of the high δ^56^Fe and unradiogenic ^187^Os/^188^Os is firmly attributed to serpentinized oceanic peridotite. The peridotite accounts for inherited unradiogenic osmium, while serpentinization accounts for the occurrence of localized, isotopically heavy, iron-rich phases. Serpentinization and its reducing conditions can also account for the reduced nature of the metallic inclusions without invoking iron disproportionation. It is envisioned that, upon being carried by subduction down to the depths of CLIPPIR diamond crystallization (360 to 750 km), the metal phase melts and may become mobile, interacting with surrounding solids to evolve into the Fe-Ni-C-S melt now seen trapped as inclusions. It is also possible that the metal interacts with a slab-derived hydrous/carbonatitic melt that could provide carbon to the metal while simultaneously oxidizing some of the iron and driving the carbon content toward saturation, similar to experiments on metal-carbonatite melt interaction (*46*). Reconstructing the details of diamond formation at depth will require further study.

Nevertheless, the metallic inclusions show that isotopically heavy, iron-rich phases in the mantle portion of the slab will be subducted, and imply that complementary isotopically light iron in serpentine and brucite (*31*) will also be subducted. These disparate isotopic signatures reside in different minerals and, as the slab warms, could remain segregated upon melting or fluid release rather than simply reequilibrating. In other words, serpentinization is accompanied by iron isotope fractionation into mineralogically distinct reservoirs that can be separated when slabs stall and warm up after sinking to the mantle transition zone. Iron derived from magnetite and/or awaruite, with the pronounced heavy iron signature (*31*) that is manifested in the metallic melt trapped in CLIPPIR diamonds, is likely to contribute to the more fusible components of the heterogeneous convecting mantle and may go on to increase the δ^56^Fe of basalts erupted at surface. In contrast, serpentine and brucite, with their slightly light iron signature (*31*), are expected to form iron-bearing Mg silicates that are more refractory mantle components. The modest light iron signature could therefore be sequestered in more refractory mantle heterogeneities.

### Implications of recycled heavy iron

Ongoing, deep subduction of serpentinized slabs, as has occurred over hundreds of millions to billions of years of plate tectonic activity, could lead to recycling-related iron isotopic heterogeneity in the convecting mantle, helping to account for the range of slightly heavy iron isotopic compositions observed in oceanic basalts (*47*). For example, if magnetite within subducted serpentinite is used as a basis to estimate the flux of recycled heavy iron with δ^56^Fe = 0.8‰, it would provide enough iron in the form of heterogeneities in the mantle sources of magmas to potentially raise the average δ^56^Fe composition of oceanic basalts by as much as 0.06‰ [assuming 10% serpentinization of the uppermost 10 km of oceanic peridotite (*2*–*4*, *8*, *31*, *48*); Supplementary Discussion]. The presence of recycled heavy iron is generally supported by studies of oceanic basalts that identify a heavy iron end-member component in the mantle associated with pyroxenites (*16*, *47*), which represent the more fusible and incompatible element–rich constituents derived from subducted oceanic lithosphere. Similarly, heavy iron isotopic signatures in ocean island basalts from Azores, Pitcairn, and Samoa are proposed to be derived from a multiplicity of mechanisms including the incorporation of heavy iron from recycled oceanic lithosphere (*17*). Our identification of a very heavy iron component in the altered mantle of subducting slabs was not explicitly considered but nonetheless supports this model.

The recycling of serpentinized peridotite in subducted oceanic lithosphere is increasingly being recognized as an important influx to the convecting mantle (*2*, *4*–*9*). Iron isotopic compositions allow tracing of the subduction-driven geochemical pathway from surface processes such as seafloor serpentinization to the depths of the mantle transition zone and lower mantle. Whereas the oceanic crust is thought to be largely devolatilized to account for arc volcanism, the slab mantle, particularly in cool slabs, remains cooler to greater depths, allowing retention of water and other surficial components in metaserpentinite (*2*, *7*). Deep serpentinite subduction is therefore supported by both the iron isotopic signature and the hydrogen (*10*, *11*) in metallic Fe-Ni-C-S inclusions in CLIPPIR diamonds, as well as the boron in sublithospheric type IIb diamonds and the hydrogen in their silicate inclusions (*5*). Oceanic serpentinites can easily supply the carbon for diamond growth, considering that they can contain up to 1500-ppm carbon with an isotopic range mirrored by sublithospheric diamonds (*41*). Thermal models of subducting slabs show that the pressure-temperature path of cool slabs will pass from the stability field of serpentine to dense hydrous magnesium silicates (*6*, *7*), aiding water and fluid mobile element retention in metaserpentinite (*2*, *6*, *49*). Water (*2*, *4*, *6*, *7*), halogens (*4*), noble gases (*8*), and other surficial materials may be deeply recycled via this pathway. The observation that subducted, metamorphosed serpentinized peridotites reach the mantle transition zone and beyond provides key information for understanding deep recycling. In turn, establishing the presence of deeply recycled heavy iron metal provides a previously unrecognized aspect for explaining mantle iron isotopic compositions inferred from oceanic basalts.

## MATERIALS AND METHODS

### Diamond samples and inclusion screening

The metallic inclusions were hosted within two different diamonds, named Letseng_889 and Letseng_890, weighing 2.39 and 6.52 carats, respectively (1 carat = 0.2 g; Fig. 1A). These are offcuts trimmed from larger diamonds during commercial manufacturing of facetted gemstones. The diamonds were recovered from the Letseng mine, Lesotho. All of the inclusions analyzed for their iron or osmium isotopic compositions were first examined using Raman spectroscopy to check for the presence of methane (fig. S1) to verify that they have remained completely sealed in the host diamond since the time of entrapment. These inclusions typically have a fluid film of methane trapped between the solid inclusion material and the walls of the diamond host (*10*, *11*), which would be released if there were any fractures reaching from the inclusion to the diamond exterior. The methane originates from hydrogen dissolved in the Fe-Ni-C-S metallic melt when it was trapped by the growing diamond. Upon cooling and solidification of the melt, the hydrogen exsolves as fluid and reacts with carbon to form methane.

Raman spectroscopy was done at the Gemological Institute of America with a Renishaw InVia Raman microscope, with 1800 lines/mm grating, using a 50×, 0.55 numerical aperture (NA) lens, and the 514.5-nm line of an argon ion laser set to 150-mW output power. The 520.5-cm^−1^ peak of a silicon standard was used for spectral calibration.

### Iron isotope geochemistry

Metallic inclusions were exposed by polishing the diamond using conventional diamond polishing methods on a scaife. After cleaning the diamond surface with distilled acetone, distilled ethanol, and Milli-Q water, a small drop of Crystalbond (an acetone-soluble, Fe-free resin with relatively low melting temperature) was applied to the diamond surface to cover the exposed metallic inclusion. The diamond fragment, with the metallic inclusion being protected by Crystalbond, was then placed in a Teflon vial and soaked in 0.4 to 3 ml of 6 M HCl at room temperature for 12 to 48 hours to further clean the diamond surface from any residual iron that might have remained from polishing on the scaife or from past handling with tweezers. The amount of iron in the cleaning acid was monitored and estimated to be within 6, 1, and 0.05% of the inclusion signals for Letseng_889 inclusion E & F, inclusion I, and Letseng_890 inclusion E, respectively. Chemical and iron isotopic compositions of potential anthropogenic contaminants, the steel polishing scaife and the stainless steel tweezers, were also measured and found to be distinct from the inclusions (table S2).

After cleaning in 6 M HCl, the Crystalbond on the diamond was removed by repeatedly washing the diamond surface with distilled acetone, distilled ethanol, and Milli-Q water. The exposed metallic inclusion was subsequently dissolved out of the diamond by bathing the whole sample in 0.4 to 1 ml of 6 M HCl at room temperature for 2 to 3 days (90°C overnight for Letseng_889 inclusion E & F). The dissolution approach was tested initially on a mineral assemblage of troilite, taenite, and cohenite that simulates the mineralogy of the metallic inclusions, and complete dissolution was achieved. After dissolution of the metallic inclusion, about 5% of the analyte solution was used to determine its Fe, Ni, V, Cr, Mn, Co, and Cu concentrations on a Thermo Fisher Scientific iCap Q quadrupole ICP-MS (inductively coupled plasma mass spectrometer) at the Carnegie Institution for Science. The rest of the original solution was used for iron isotopic analyses.

Iron was purified from the matrix using one-step ion-exchange chromatography on heat-shrink Teflon columns loaded with 0.1 ml of Bio-Rad AG 1-X8 resin (100 to 200 mesh). The sample was loaded in 50 μl of 6 M HCl + 0.001% H_2_O_2_. After removing the matrix by eluting 1.2 ml of 6 M HCl + 0.001% H_2_O_2_ through the column, the Fe fraction of the sample was eluted from the resin in 2 ml of 0.1 M HCl. The purified Fe solution was evaporated to dryness, taken up in 5 μl of concentrated HNO_3_ and 400 μl of concentrated H_2_O_2_, and then left at ~90°C overnight to decompose eluted organic compounds from the resin. The sample was then evaporated to dryness again and taken up in 1 ml of 0.4 M HNO_3_ for iron isotopic analyses. The column procedure gives an Fe yield of ~100%. Because of the use of concentrated H_2_O_2_, a small percentage of Fe could occasionally be lost during evaporation, making the total yield 90 to 100%. To ensure that there was no isotopic fractionation when that occurred, we performed multiple rounds of calibrations to show that there was no measurable effect (table S1). The procedural blank for Fe is less than 1 ng and negligible compared to the amount of Fe in the samples.

Iron isotopic compositions of purified samples were analyzed using a Nu Plasma II MC-ICP-MS (multicollector ICP-MS) at the Carnegie Institution for Science. Sample solutions were diluted to 200 parts per billion (ppb) in concentration and introduced to the mass spectrometer in dry plasma mode using an Aridus 3 desolvating nebulizer. Measurements were performed in high-resolution mode to resolve Fe^+^ peaks from isobaric interferences of ArN^+^, ArO^+^, and ArOH^+^. Potential isobaric interference from ^54^Cr^+^ was monitored at mass ^53^Cr^+^ and subtracted from Fe signals as necessary. The monitored intensity on ^53^Cr^+^ is <1 mV, and the correction on ^54^Fe^+^ is within 0.3 mV, insignificant to cause any measurable fractionation as tested on isotope standards. The instrument mass fractionation was corrected using the standard-sample bracketing method with the peak intensities matched to <10% difference. Each sample was analyzed four to eight times, with each analysis including 20 cycles of 4-s integrations. A sensitivity of 45 to 90 V/ppm (i.e., 9 to 18 V for a 200-ppb solution) was achieved for ^56^Fe. Iron isotopic compositions are reported relative to IRMM-524a, which has the same iron isotopic composition as the international standard IRMM-014 (*50*)

δ56Fe (‰)=[(Fe56/Fe54)Sample(Fe56/Fe54)IRMM−524a−1]×1000

To check the accuracy of the MC-ICP-MS analyses, three geological standards (BHVO-2, BIR-1, and AGV-2) were purified following the “short-column” procedure of Craddock and Dauphas (*50*) using 1 ml of AG 1-X8 resin and analyzed under the dry plasma mode. Measured iron isotopic compositions of the three geological standards agree well with literature data (table S1). To test the column procedure used in this study, a troilite-taenite-cohenite mineral assemblage (TTC1) was dissolved and purified following both the well-established short-column method of Craddock and Dauphas (*50*) and the 0.1-ml heat-shrink Teflon column method developed in this study for comparison. Both methods yielded consistent results of ~0.1‰ within analytical error (table S1). The tests using the 0.1-ml heat-shrink Teflon column method showed the ability to process samples containing as little as 0.2 μg of Fe without causing measurable isotope fractionation (table S1), which is several times lower than the amount of iron recovered from the inclusions. In addition to the geological standards and the synthetic standard, 0.2 μg of the Fe isotope standard (IRMM-524a) was doped with Ni, Co, Cr, and Cu and purified using the 0.1-ml columns. An isotopic composition of ~0.0‰ was obtained for the product, indicating negligible isotopic fractionation during the column procedure (table S1).

### Osmium isotope geochemistry

Osmium isotope measurements for two metallic inclusions in sample Letseng_889 (inclusion J and inclusion K) were made on a Thermo Fisher Scientific Triton thermal ionization mass spectrometer at the Carnegie Institution for Science using the same microchemical extraction and negative thermal ionization techniques as used in studies of sulfide inclusions in lithospheric diamonds (*51*, *52*). The inclusions were partly exposed by cleaving open the host diamond. The multiphase assemblage of each inclusion was then characterized by qualitative energy-dispersive x-ray spectroscopy (EDS) analysis (Fig. 1B). To facilitate handling of these 50- to 100-μm inclusions, which are highly susceptible to electrostatic/magnetic forces, Os was dissolved and distilled directly from the inclusions while they were still attached to the cleaved diamond surface. Re and Os concentrations for the inclusions could not be accurately determined for practical reasons. Their weights were difficult to estimate while attached to the diamond, and their Os contents were much higher than expected. The resulting sample solutions were critically underspiked. The very high isotope dilution error magnification (EM) applicable to these sample-spike mixtures meant that measured isotope dilution ratios were within EM-propagated error of the normal (unspiked) isotopic composition. Under such circumstances, the ability to determine precise Os isotopic compositions is unaffected because all that is being measured is the normal Os in the sample (i.e., negligible spike Os correction). In addition, the chromic-sulfuric reagent preferentially attacks the pyrrhotite, and its dissolution efficacy on Fe-Ni metal, cohenite, and other accessory phases such as Cr-Fe oxide is not known. Since the separation of the initially homogeneous Fe-Ni-C-S melt during cooling into pyrrhotite, cohenite, and Fe-Ni metal will fractionate Re from Os differently in each phase, actual concentrations and Re/Os ratio would have been impossible to interpret. Again, this effect does not hinder the measured Os isotopic compositions.

### NRIXS analysis

Room-pressure, room-temperature NRIXS analysis was conducted at sector 3-ID-B of the Advanced Photon Source (APS) at Argonne National Laboratory. Energy spectra were obtained from −90 to +130 meV around the elastic peak with a step size of 0.25 meV and a collection time of 3 s per step. A total of 20 scans were conducted for the sample. A millimeter-size piece of awaruite from Josephine County in Oregon (NMNH 106125) was sliced and polished to expose a fresh surface for NRIXS analyses. The awaruite slice was checked under scanning electron microscopy for impurities before the analyses. Data reduction was done with the software packages PHOENIX (*53*) and SciPhon (*54*), which yielded similar results.

## SUPPLEMENTARY MATERIALS

Supplementary material for this article is available at http://advances.sciencemag.org/cgi/content/full/7/14/eabe9773/DC1
